# Mutations in *SCG10* Are Not Involved in Hirschsprung Disease

**DOI:** 10.1371/journal.pone.0015144

**Published:** 2010-12-20

**Authors:** Maria M. M. Alves, Jan Osinga, Joke B. G. M. Verheij, Marco Metzger, Bart J. L. Eggen, Robert M. W. Hofstra

**Affiliations:** 1 Department of Genetics, University Medical Center Groningen, University of Groningen, Groningen, The Netherlands; 2 Translational Centre for Regenerative Medicine, University of Leipzig, Leipzig, Germany; 3 Department of Neuroscience, Section Medical Physiology, University Medical Center Groningen, University of Groningen, Groningen, The Netherlands; Health Canada, Canada

## Abstract

Hirschsprung disease (HSCR) is a congenital malformation characterized by the absence of enteric neurons in the distal part of the colon. Several genes have been implicated in the development of this disease that together account for 20% of all cases, implying that other genes are involved. Since HSCR is frequently associated with other congenital malformations, the functional characterization of the proteins encoded by the genes involved in these syndromes can provide insights into the protein-network involved in HSCR development. Recently, we found that KBP, encoded by the gene involved in a HSCR- associated syndrome called Goldberg-Shprintzen syndrome, interacts with SCG10, a stathmin-like protein. To determine if SCG10 is involved in the etiology of HSCR, we determined SCG10 expression levels during development and screened 85 HSCR patients for SCG10 mutations. We showed that SCG10 expression increases during development but no germline mutation was found in any of these patients. In conclusion, this study shows that SCG10 is not directly implicated in HSCR development. However, an indirect involvement of SCG10 cannot be ruled out as this can be due to a secondary effect caused by its direct interactors.

## Introduction

Hirschsprung disease (HSCR) is an abnormality of the enteric nervous system characterised by the lack of ganglia along a variable length of the gut. It is a developmental disorder that arises due to failure in migration of enteric neural crest cells into the intestinal tract or due to a failure in survival, proliferation, or correct development of enteric neurons once they reach the gut [1]. In either case an aganglionic segment occurs in which contraction and relaxation is absent. The length of the aganglionic segment can vary and HSCR patients are classified into short segment (S-HSCR, 80%), long segment (L-HSCR, 15%), or total colonic aganglionosis (TCA, 5%) [2]. HSCR is a common genetic disorder with an estimated incidence of 1∶5000 live births and is more frequent in males than in females (4∶1), a difference most prominent in S-HSCR [3].

Several genes have been implicated in the development of this disease, being *RET* the most important one [4]. *RET* (REarranged during Transfection) encodes a receptor tyrosine kinase which is expressed in neural crest derived lineages, playing a pivotal role during development of the enteric nervous system [1]. Mutations in the coding region of *RET* are responsible for 50% of familial HSCR cases and 15% of sporadic ones [5]. However, they only account for 20% of all HSCR cases, suggesting that other genes are involved. Since HSCR is frequently associated with other congenital malformations present in rare recessive syndromes, the study of the proteins involved in these syndromes can bring new insights about HSCR development. Such syndrome, in which HSCR is frequently observed, is Goldberg-Shprintzen syndrome (GOSHS), a rare autosomal recessive disorder. The gene responsible for this syndrome is *KBP* and recently we identified the Superior Cervical Ganglia Neural Specific-10 or SCG10 as the major interacting protein of KBP [6], [7]. SCG10 is a stathmin-like protein involved in microtubule dynamics [8]. But unlike other stathmins, SCG10 acts in two ways to promote microtubule dynamics: it stabilizes microtubules at their plus end and promotes microtubule catastrophe at their minus end [9]. Moreover, SCG10 is known to play an important role in neuronal differentiation by enhancing neurite outgrowth, a phenomenon that is believed to be dependent on microtubule dynamics [8]. Also, *SCG10* has been previously identified as a down-regulated gene in a RET mouse model for HSCR [10] and we have shown that SCG10 interacts with KBP *in vitro* and *in vivo* [7]. All together these facts suggest that SCG10 might also play a role in HSCR development.

In this paper we evaluate the role of SCG10 in HSCR development by determining SCG10 expression levels during the process of gut colonization and by screening a set of isolated non-syndromic HSCR patients without *RET* mutations for the presence of *SCG10* mutations.

## Results

### Expression of SCG10 in enteric neural crest stem cells (ENCSCs)

**Figure 1:**
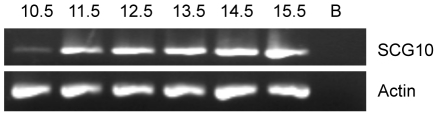
SCG10 expression levels during the development of the enteric nervous system. Expression studies in mouse enteric neural crest cells show that RNA levels of SCG10 are upregulated in the initial stage of gut colonization [11.5] and remain constant until the whole process is finished by E15.5.

To determine SCG10 involvement in the development of the enteric nervous system, SCG10 expression levels were analysed in ENCSCs. Because gut colonization occurs between day 9 and 15.5 of mouse development, ENCSCs were isolated from E10.5 until E15.5 mouse and maintained for 2 weeks in an undifferentiated state. In these ENCSCs, SCG10 expression was detected in the earliest stage analysed, E10.5, and an increase in expression levels was observed in the following stages (Fig. 1). Our data shows that there is an up-regulation of SCG10 expression levels in ENCSCs.

### Mutation screening of SCG10

To further evaluate the involvement of SCG10 in HSCR development, we screened blood DNA from 85 patients diagnosed with isolated, non-syndromic HSCR. No germline mutation was detected in *SCG10*.

## Materials and Methods

### Culture of ENCSCs

ENCSCs were prepared from embryonic gut (jejunum to rectum) of C57BL/6 mice E10.5, 11.5, 12.5, 13.5, 14.5 and 15.5. The gut was mechanically dissected and then tissues were incubated in collagenase/dispase enzyme solution (collagenase XI [750 U/ml; Sigma-Aldrich] and dispase II [250 µg/ml; Roche]) in PBS for approximately 5 minutes at 37°C. Digested tissue was triturated until a homogenous cell suspension was created. Cell suspensions were washed and seeded on 35-mm petri dishes previously coated with fibronectin (2 µg/cm^2^, Sigma-Aldrich). DMEM-F12 medium (PAA) containing N2 supplement (Invitrogen), B27-supplement (Invitrogen), 1% penicillin/streptomycin (PAA), 20 ng/ml fibroblast growth factor (FGF, Peprotech) and 20 ng/ml epidermal growth factor (EGF, Peprotech), was used as culture medium. Every two days half of the medium was replaced with new medium containing freshly added FGF and EGF. The enteric neural crest-derived cells were kept in culture as neurospheres-like bodies (NLBs) for 14 days.

### RNA extraction and RT-PCR

After 14 days in culture, NLBs were collected and washed with PBS. RNA extraction was performed using the RNeasy mini kit^©^ (Qiagen). First strand cDNA was originated from 2 ug of RNA using the Ready-To-Go You-Prime First-Strand Beads^©^ kit (GE Healthcare) and pdN[6] as first-strand primer. First-strand cDNA generated by this method was directly used for RT-PCR. The primers used for amplification were SCG10 forward (5′-ACAATGGCTAAAACAGCAATGGC-3′) and SCG10 reverse (5′-TGCTTCAGCCAGAC-3′), Actin forward (5′-ATATCGCTGCGCTGGTCGTC-3′) and Actin reverse (5′-AGGATGGCGTGAGGGAGAGC -3′).

### Patients

All patients included in this study were diagnosed with HSCR disease and showed no mutations in the *RET* gene. All patients included in this study gave their written informed consent. Ethical approval was obtained from the UMCG ethical committee (Medisch Ethische Toetsings commissie-UMCG). In total, 85 patients were screened. From these 85 patients 43 were S-HSCR, 11 were L-HSCR and for 31 cases the length of the segment affected was unknown.

### Mutation screening of SCG10

Genomic DNA was isolated from peripheral blood lymphocytes by use of standard methods. Sequencing of exons 1-5 of *SCG10* was performed using 5 sets of primers (for primers and PCR conditions see table 1). DNA amplification was performed using 50 ng of genomic DNA in 30 µl PCR reactions containing: 10X reaction buffer, 10 µM primer pair mix, 25 mM dNTPs and 1 unit Taq Polymerase (Amersham). PCR products were amplified and purified (ExoSap it – GE Healthcare). Direct sequencing of exons 1–5 was performed using the 3730 DNA Analyser (Applied Biosystems). Using the software Mutation Surveyor (Version 3.23, SoftGenetics LLC) sequences were aligned and compared with consensus sequences.

**Table 1:** PCR conditions.

Primer Sequences	bp	Size(bp)	Tm °C PCR	Seq.prim
SCG10 1F TCTAGCACGGTCCCACTCTG	20	174	58	1F
SCG10 1R AGGTAGAGCCGACGGAGAAC	20			
SCG10 2F ACCTGGCAATATTCACTCTG	20	340	58	2F
SCG10 2R TAGACACGGCAAGTCAATAG	20			
SCG10 3F CTCCCGGAATAACAACGCTAC	21	444	58	3F
SCG10 3R ACATGTTGGCATGGCACAG	19			
SCG10 4F CCGTTATTCTGCTAGGTTTG	20	327	58	4F
SCG10 4R TCAGGCATATGGAAGTTCAC	20			
SCG10 5F TAGACACCAAACTGGGTTAC	17	236	58	5F
SCG10 5R ATCCTGATATCGCATGATCC	20			

## Discussion

Hirschsprung disease (HSCR) is a developmental disorder in which the process of gut colonization is disturbed. To date, 11 genes have been reported to be involved in HSCR development: *RET*, *GDNF*, *NTN*, *EDNRB*, *EDN3*, *SOX10*, *ECE-1*, *ZFHX1B*, *PHOX2B*, *KBP and NRG1* [4], [6], [11]–[19]. However, all together, they only explain approximately 20% of all HSCR cases. To better understand HSCR development, the study of rare syndromes that are characterized by the presence of HSCR can be of major importance, as determining the protein network connected to the mutated proteins can help identifying candidate genes. In this study we focus on such a candidate gene, *SCG10*, encoding a microtubule destabilizing protein, recently described to interact with KBP and believed to be important in the development of Goldberg-Shprintzen syndrome and consequently, in HSCR [7]. Furthermore, *SCG10* has been previously identified as a downregulated gene in a RET mouse model for HSCR [10] reinforcing the idea that SCG10 might also play a role in HSCR development. Our results showed that SCG10 expression is clearly up-regulated during the process of gut colonization, which suggests that SCG10 migth play a role in the development of the enteric nervous system. However, *SCG10* screening in a set of 85 patients diagnosed with isolated, non-syndromic HSCR showed that no mutation was present. Taken together, these results suggest that although SCG10 seems necessary for the development of the enteric nervous system, it is not directly implicated in HSCR. A possible explanation for this result is that SCG10 involvement in HSCR can be a consequence of a disruption in the normal protein network necessary for proper development. SCG10 activity is known to be controlled by two post-translational modifications: palmitoylation and phosphorylation [20]. The first one is responsible for growth cone targeting of SCG10 and the second one is responsible for controlling SCG10 activity. As RET is a tyrosine kinase receptor known to be involved in several signalling pathways, it is possible that RET, via one of its downstream pathways, controls SCG10 activity, linking this gene directly to HSCR. Further studies are required to determine if there is any association between RET and SCG10 and a possible involvement of SCG10 in HSCR development.
